# Identification of Auditory Object-Specific Attention from Single-Trial Electroencephalogram Signals via Entropy Measures and Machine Learning

**DOI:** 10.3390/e20050386

**Published:** 2018-05-21

**Authors:** Yun Lu, Mingjiang Wang, Qiquan Zhang, Yufei Han

**Affiliations:** Key Laboratory of Shenzhen Internet of Things Terminal Technology, Harbin Institute of Technology Shenzhen Graduate School, Shenzhen 518055, China

**Keywords:** auditory attention, entropy measure, linear discriminant analysis (LDA), support vector machine (SVM), auditory attention classifier, electroencephalography (EEG)

## Abstract

Existing research has revealed that auditory attention can be tracked from ongoing electroencephalography (EEG) signals. The aim of this novel study was to investigate the identification of peoples’ attention to a specific auditory object from single-trial EEG signals via entropy measures and machine learning. Approximate entropy (ApEn), sample entropy (SampEn), composite multiscale entropy (CmpMSE) and fuzzy entropy (FuzzyEn) were used to extract the informative features of EEG signals under three kinds of auditory object-specific attention (Rest, Auditory Object1 Attention (AOA1) and Auditory Object2 Attention (AOA2)). The linear discriminant analysis and support vector machine (SVM), were used to construct two auditory attention classifiers. The statistical results of entropy measures indicated that there were significant differences in the values of ApEn, SampEn, CmpMSE and FuzzyEn between Rest, AOA1 and AOA2. For the SVM-based auditory attention classifier, the auditory object-specific attention of Rest, AOA1 and AOA2 could be identified from EEG signals using ApEn, SampEn, CmpMSE and FuzzyEn as features and the identification rates were significantly different from chance level. The optimal identification was achieved by the SVM-based auditory attention classifier using CmpMSE with the scale factor *τ* = 10. This study demonstrated a novel solution to identify the auditory object-specific attention from single-trial EEG signals without the need to access the auditory stimulus.

## 1. Introduction

Existing relevant research has revealed that auditory objects [1], as neural representational units encoded in the human auditory cortex [2], are involved with high-level cognitive processing in the cerebral cortex, such as top-down attentional modulation [3]. Top-down attention is a selection process that focuses cortical processing resources on the most relevant sensory information in order to enhance information processing. There are many reports that auditory attention can be detected from brain signals, such as invasive electrocorticography [4], non-invasive magnetoencephalography (MEG) [5,6] and electroencephalography (EEG) [7,8]. These findings provide hard evidence that peoples’ attention to a specific auditory object, which is referred to as the auditory object-specific attention, can be identified from brain signals. As the reflection of electrical activity in the cerebral cortex, EEG signals contain a wealth of information which is closely relating to advanced nervous activities in human brain such as learning, memory and attention [9]. Owing to the advantages of relatively low cost, easy to access and high temporal resolution, EEG signals are of much more practical value for the study of auditory object-specific attention [10].

In recent years, many attempts have been made to identify auditory object-specific attention from EEG signals. According to a review of the available literature, there are mainly three approaches to achieving the identification of auditory object-specific attention from ongoing EEG signals:

- The first approach is the use of system identification, which is mainly to build a linear forward map from the auditory stimuli of specific acoustic features on EEG signals. This is a direct method to estimate EEG signals [11]. The auditory object-specific attention can be inferred from the estimated EEG signals [5].
- The second approach is the use of stimulus reconstruction, which is mainly to reconstruct specific acoustic features (temporal envelopes) of auditory stimuli from the ongoing EEG signals [12]. Recently, this classical method has been extensively used to study the processing of speech perception in EEG signals. The auditory object-specific attention can be identified based on the reconstructed the acoustic features [7].
- The third approach is to extract the informative features of EEG signals and/or auditory stimuli and then exploit machine learning algorithms to train a classifier for the detection of auditory attention [10,13]. The informative features can be the cross-correlation between EEG signals and an auditory stimulus’ envelope, the power in EEG signal bands, the measure of auditory event-related potentials [14] and so on. Machine learning algorithms, such as linear discriminant analysis (LDA) [15], regularized discriminant analysis (RDA) [13], support vector machine (SVM) [16,17], neural networks [18] and so on, have been reported in the published studies.

According to the available research, the first and second approaches must exploit EEG signals and acoustic features of auditory stimulus to achieve the identification of auditory object-specific attention. The auditory attention identification from EEG signals usually requires that the acoustic features of auditory stimuli be known in advance, for instance, the speech envelopes in the study of Horton [15]. Because the identification of auditory attention from EEG signals were based on that the cortical oscillations phase locked to the envelope of the auditory stimuli [19] and the temporal envelope of the auditory stimuli could be reconstructed from individual neural representations [2]. However, machine learning techniques recently have made remarkable progress and can achieve unprecedented accuracy for classification tasks [20]. With the help of machine learning, the third approach has the potential to achieve the detection of auditory object-specific attention by exploiting the enough useful information from EEG signals alone.

Using entropy measures to extract the informative features of EEG signals for brain-state monitoring and brain function assessment are becoming the hot research topics. EEG signals are commonly accepted to be non-stationary, nonlinear and multicomponent in nature. As typical nonlinear analysis methods, entropy measures in EEG signals may be much more appropriate to capture the imperceptible changes in different physiological and cognitive states of human brain. For example, Mu et al. studied the detection of driving fatigue using four entropy measures, i.e., spectrum entropy, approximate entropy (ApEn), sample entropy (SampEn) and fuzzy entropy (FuzzyEn), to extract features of EEG signals and reached an average recognition accuracy of 98.75% [21]. Hosseini et al. proposed the use of ApEn and wavelet entropy in EEG signals for emotion state analysis and the research found that ApEn and wavelet entropy were capable of discriminating emotional states [22]. Shourie et al. adopted ApEn to investigate the differences between EEG signals of artists and non-artists during the visual perception and mental imagery of some paintings and at resting condition. The research found that ApEn was significantly higher for artists during the visual perception and the mental imagery when compared with nonartists [23,24]. Alaraj and Fukami exploited ApEn to quantitatively evaluate the wakefulness state and the results showed that ApEn outperformed other conventional methods with respect to the classification of awake and drowsy subjects [25]. To date, applying entropy measures to the quantification of EEG signals has been proved to be a powerful tool to identify mental tasks and reveal cerebral states.

In continuation of the aforementioned studies, we move one step ahead in this study and explore the feasibility of using entropy measures on EEG signals to identify auditory object-specific attention, without the need for the acoustic features of auditory stimulus. Using four well-established entropy measures, i.e., ApEn, SampEn, composite multiscale entropy (CmpMSE) and FuzzyEn, to extract the informative features of EEG signals, we investigate the changes of these entropy measures in EEG signals under different auditory object-specific attention. Then, we use machine learning to train an auditory attention classifier for the identification of auditory object-specific attention. Based on preliminary experiment research, we demonstrate a novel solution to the identification of auditory object-specific attention from the ongoing EEG signals by the use of auditory attention classifier. The study of identification of auditory object-specific attention not only has great research value on monitoring the cognitive and physiological states of human brain, but also has great potential of the realization of assistive hearing technology with neural feedback.

## 2. Methods

### 2.1. Subjects

Thirteen subjects (aged 21 to 28 years, four females) participated in this study. All subjects were normal-hearing and right-handed college students and none had a history of neurological illness, which were confirmed by questionnaires. The experiment procedures were approved by the ethics committee of Harbin Institute of Technology Shenzhen Graduate School and all experiments were performed in accordance with relevant guidelines and regulations. Informed consent forms were signed by the subjects before the experiments were performed.

### 2.2. Experimental Design

In the study two audio signals were used as the auditory stimuli and the durations of both the audio signals were 60 s. While the subjects focused their attention on listening to the specific auditory stimulus, the corresponding specific auditory object was emerging in their auditory cortex. The audio signal A was the roaring sound of tiger corresponding to the Auditory Object1; the audio signal B was a segment of a stand-up comedy corresponding to the Auditory Object2. The audio signals were binaurally played with ER4 (Etymotic Research Inc., Elk Grove Village, IL, USA) in-ear earphones. The subjects were instructed to keep their attention on the auditory stimuli with their eyes closed while their scalp EEG signals were being recorded.

In this study 8-channel EEG signals were recorded using the ENOBIO 8 system (Neuroelectrics, Barcelona, Spain) with dry electrodes. The EEG signals were sampled at 500 Hz with band pass filter 0.540 Hz from eight sites on the scalp. According to the international standard 10–20 system, the eight electrode sites were selected to be T7 and T8 in the temporal region, P7 and P8 in the posterior temporal region, P3 and P4 in the parietal region, Cz in the central region and Fz in the frontal region, respectively. In order to minimize the possibility of the movements of subject’s body as much as possible during experiment, the subjects were asked to sit in a comfortable chair.

The experiments were conducted in a soundproof room. Each subject was required to undergo three EEG measurement protocols in a random order and there were totally 39 EEG measurements in this study, each of 60 s in length. The three 60-s EEG measurement protocols corresponded to three kinds of auditory object-specific attention. The first EEG measurement protocol, during which the subject was instructed to keep calm and his brain was in a resting state without any audio signal playing, corresponded to Rest. The second EEG measurement protocol, during which the subject was instructed to keep his attention on the auditory stimulus with the audio signal A playing, corresponded to Auditory Object1 Attention (AOA1). The third EEG measurement protocol, during which the subject was instructed to keep his attention on the auditory stimulus with the audio signal B playing, corresponded to Auditory Object2 Attention (AOA2). For each subject the three EEG measurement protocols were randomly performed to reduce EEG signals to be contaminated by a fixed order of auditory tasks or the dominance of ears.

### 2.3. Entropy Measures in EEG Signals

ApEn, proposed by Pincus [26], is considered as a complexity measure of time series. ApEn has the ability of measures of predictability based on evaluating the irregularity of time series. The more similar patterns the time series has, the less irregular the time series are and the more likely the time series are to be predictable. The computation method of ApEn is firstly involved in the phase space reconstruction, in which the time series are embedded into phase spaces of dimension *m* and *m* + 1, respectively; and then calculates the percentages of similar vectors in phase spaces with acceptable matches. SampEn, proposed by Richman et al. [27], is a modified version of ApEn. SampEn has better performance over ApEn in the consistency and dependence on data length. CmpMSE was proposed by Wu [28]. For a predefined scale factor, a set of *k-*th coarse-grained time series based on the composite averaging method are reconstructed from original time series. The sample entropies of all coarse-grained time series are calculated, and then CmpMSE is defined as the mean of all the sample entropies. FuzzyEn used the fuzzy membership function to obtain a fuzzy measurement of two vectors’ similarity [29]. The family of exponential function was usually used as the fuzzy function and it was continuous and convex so that the similarity does not change abruptly [30].

Before the calculation of ApEn, SampEn, CmpMSE and FuzzyEn, EEG signals were preprocessed by linear detrending, which the polynomial curve fitting were used as the trend terms of EEG signals and then subtracted. Then, a 9-level wavelet decomposition was performed, using Daubechies (db4) wavelets as the wavelet function which was suitable for detecting changes of the EEG signals [31]. Two of the highest detail coefficients D2 (62.5–125 Hz) and D1 (125–250 Hz) [31] and the approximation coefficients A9 (0–0.49 Hz) were considered as noises. The denoised EEG signals were recovered by the detail coefficients from D3 and D9 and the effective frequency band of the denoised EEG signals was considered to be 0.5–62.5 Hz. The recording time of EEG signals was 60 s, which corresponded to the durations of both the audio stimuli. Because of the sampling rate was 500, the data length used for the calculation of entropy measures in EEG signals was *N* = 30,000. For the calculation of ApEn, SampEn and FuzzyEn, the embedding dimension *m* = 2 and the tolerance *r* = 0.15 × STD, where STD was the standard deviation of EEG signals. For the calculation of CmpMSE, the scale factor was selected to *τ* = 30 and the parameter *m* and *r* were the same as ApEn and SampEn. The assignment of the parameters for the calculation of ApEn, SampEn, CmpMSE and FuzzyEn, are shown in Table 1.

### 2.4. Statistical Analysis Methods

The statistical analysis methods included the multiple-sample tests for equal variances, Shapiro-Wilk W test for normal distribution, parametric or non-parametric analysis of variance and multiple comparisons, which were used to evaluate ApEn, SampEn, CmpMSE and FuzzyEn in EEG signals under auditory object-specific attention of Rest, AOA1 and AOA2. The multiple-sample tests for equal variances were to use the Bartlett null hypothesis test that the values of the entropy measure in EEG signals under examination had the same variance. The Shapiro-Wilk W test was used to examine normality of the values of ApEn, SampEn, CmpMSE and FuzzyEn under the null hypothesis that the values of the entropy measures under examined obeyed normal distributions. If the values of the entropy measures in EEG signals under auditory object-specific attention of Rest, AOA1 and AOA2 had equal variances and obeyed normal distributions, the parametric analysis of variances and multiple comparisons were performed to determine whether the values of the entropy measures were significantly different from each other for Rest, AOA1 and AOA2 of auditory object-specific attention; otherwise, the Kruskal-Wallis test (an extension of the Wilcoxon rank sum test) and multiple comparisons were used. Besides, Bonferroni correction was used to counteract the problem of multiple comparisons.

### 2.5. Auditory Attention Classifier Based on Entropy Measures and Machine Learning

Machine learning, which learns from data to make data-driven predictions or decisions, is now widely used for the analysis of EEG signals [32]. Machine learning is the method that is used to train a model and develop related algorithms with the input features and lend itself to prediction. In this study three kinds of auditory object-specific attention were investigated, i.e., Rest, AOA1 and AOA2. To demonstrate auditory attention classifier, we used ApEn, SampEn, CmpMSE and FuzzyEn to extract the information of EEG signals as features and then exploited LDA and SVM to construct the auditory attention classifiers. As a classical machine learning method, LDA is a statistical classifier which achieves a linear decision boundary based on the within and between class scatter matrices [33]. LDA performs the discrimination of different classes by maximizing the between class scatter and minimizing the within class scatter. LDA has been commonly used for EEG signals classification, which allows for fast and massive processing of data samples [34,35]. Like LDA, SVM also is one of the most machine learning methods, which can be not only used for the linear classification but also for the non-linear classification using a specific kernel function.

The thirteen subjects’ EEG signals were recorded and each subject underwent three EEG measurement protocols. Therefore, a total of 39 samples were available for the auditory attention classifier. To facilitate the training and testing of the auditory attention classifier, LDA and SVM are employed for supervised learning. After training, the LDA-based and SVM-based auditory attention classifiers were capable of identifying the auditory object-specific attention of Rest, AOA1 and AOA2. In order to assess the identification of auditory object-specific attention, we used the leave-one-out cross-validation (LOOCV) approach to examine the identification accuracy of auditory object-specific attention of Rest, AOA1 and AOA2.

## 3. Results

### 3.1. Statistical Results of Entropy Measures in EEG Signals

According to the statistical results of multiple-sample tests for equal variances, the values of ApEn, SampEn, CmpMSE and FuzzyEn all obeyed equal variances (all *p* > 0.05) with respect to Rest, AOA1 and AOA2 of auditory object-specific attention. According to the statistical results of Shapiro-Wilk W test, for ApEn and CmpMSE in EEG signals under auditory object-specific attention of Rest, AOA1 and AOA2, the vast majority of cases of ApEn and CmpMSE obeyed normal distributions (*p* > 0.05), except in the case of ApEn in EEG signals with P8 channel (*p* = 0.012) under the auditory object-specific attention of AOA2, and except in the case of CmpMSE in EEG signals with Fz channel (*p* = 0.031) under auditory object-specific attention of AOA1. For SampEn in EEG signals under auditory object-specific attention of Rest, AOA1 and AOA2, the majority of cases of SampEn obeyed normal distributions, except in the case of SampEn in EEG signals with Fz channel (*p* = 0.038) under auditory object-specific attention of Rest and with Cz, Fz and P3 channels (*p* = 0.036, 0.013 and 0.037, respectively) under auditory object-specific attention of AOA2. For FuzzyEn in EEG signals under auditory object-specific attention of Rest, AOA1 and AOA2, the vast majority of cases of FuzzyEn obeyed normal distributions, except in the case of FuzzyEn in EEG signals with T7, T8 and P8 channels (*p* = 0.013, 0.005 and 0.0003, respectively) under auditory object-specific attention of AOA2.

Thus, according to the normality and non-normality of these entropy measures, the parametric and non-parametric analysis of variance were carried out respectively to test the significance of difference degree of these entropy measures among Rest, AOA1 and AOA2. For ApEn of P8 channel, the values of ApEn in EEG signals did not obey normal distribution, so the Kruskal-Wallis test was carried out; but for ApEn of the other channels, the values ApEn in EEG signals obeyed normal distributions, so the one-way analysis of variance was carried out. Like ApEn, for SampEn, CmpMSE and FuzzyEn the same statistical methods were applied to test the significance of difference degree of these entropy measures among Rest, AOA1 and AOA2.

**Figure 1:**
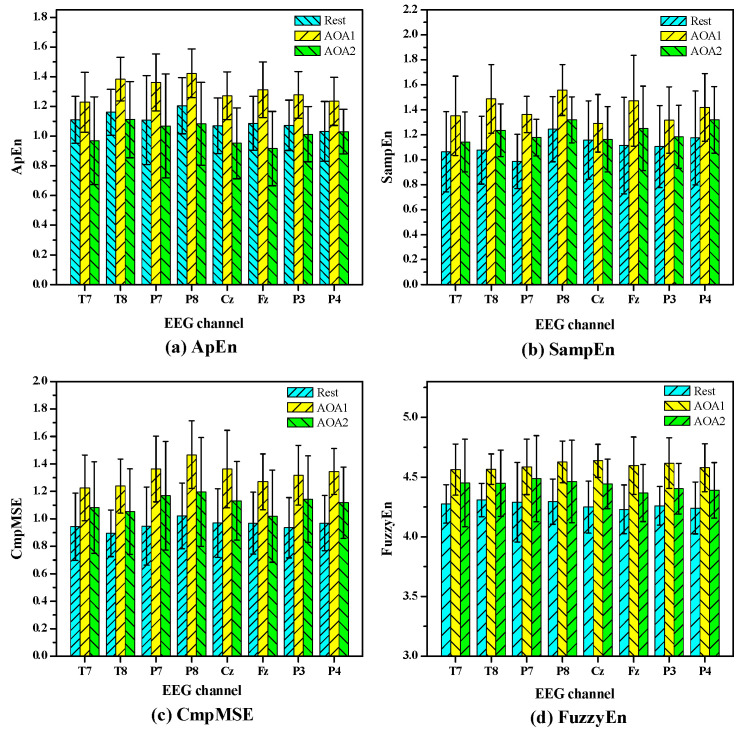
The statistical results of the values of the entropy measures in EEG signals under auditory object-specific attention of Rest, Auditory Object1 Attention (AOA1) and Auditory Object2 Attention (AOA2). (**a**) ApEn; the *p* values of eight channels are 0.021, 0.002, 0.028, 0.001, 0.001, 0.001, 0.001 and 0.006, corresponding to T7, T8, P7, P8, Cz, Fz, P3 and P4, respectively; (**b**) SampEn; the *p* values of eight channels are 0.049, 0.01, 0.001, 0.002, 0.213, 0.017, 0.105 and 0.142, respectively; (**c**) CmpMSE; the *p* values of eight channels are 0.045, 0.003, 0.007, 0.002, 0.003, 0.009, 0.002 and 0.001, respectively; (**d**) FuzzyEn; the *p* values of eight channels are 0.021, 0.005, 0.051, 0.004, 0.0001, 0.0005, 0.0001 and 0.0009, respectively. The values of these entropy measures are given as means ± standard errors. The small *p* value of 0.05 indicates that the values of the entropy measures among Rest, AOA1 and AOA2 significantly differ.

Figure 1 shows the statistical results of the values of ApEn, SampEn, CmpMSE and FuzzyEn in EEG signals under auditory object-specific attention of Rest, AOA1 and AOA2. The values of these entropy measures are given as means ± standard errors. The *p* value denotes the levels of significance of the difference of group means and the small *p* value of 0.05 indicates that the values of the entropy measures among Rest, AOA1 and AOA2 significantly differ (*p* < 0.05). For ApEn and CmpMSE, the all *p* values of eight channels are less than 0.05 and indicate that there are significant differences (*p* < 0.05) of ApEn and CmpMSE in EEG signals under auditory object-specific attention of Rest, AOA1 and AOA2. For SampEn the *p* values of T7, P7, T8, P8 and Fz channels are less than 0.05 and the other channels’ *p* values are greater than 0.05. For FuzzyEn the *p* values of most channels are less than 0.05, except for P7 channel.

To further investigate which pairs of means were significantly different for Rest, AOA1 and AOA2, the multiple comparisons test was carried out. Because there were three kinds of auditory object-specific attention, for multiple comparisons test each entropy measure was testing three (3 × 2/2) independent hypotheses. Therefore, here a *p* value of <0.015 (0.05/3) was considered statistically significant using the Bonferroni criterion. Table 2 shows the *p* values of multiple comparisons between the auditory object-specific attention of Rest, AOA1 and AOA2. In Table 2, it is clearly observed that there are significant differences in ApEn of T8, P8, Cz, Fz, P3 and P4 channels between AOA1 and AOA2, in ApEn of P3 and P4 channels between Rest and AOA1; in SampMSE of T8, P7, P8 and Fz channels between Rest and AOA1; in CmpMSE of T8, P7, P8, Cz, Fz, P3 and P4 channels between Rest and AOA1; in FuzzyEn of T7, T8, P8, Cz, Fz, P3 and P4 channels between Rest and AOA1, in FuzzyEn of Cz, Fz, P3 and P4 channels between AOA1 and AOA2. It must be noted that, for SampEn of Cz, P3 and P4 channels and FuzzyEn of P7 channel, there was no need to perform the multiple comparisons because there were no significant differences in SampEn of Cz, P3 and P4 channels and FuzzyEn of P7 channel among Rest, AOA1 and AOA2 for the parametric or non-parametric analysis of variance.

As shown in Figure 1, it is clearly observed that there are obvious differences in the mean values of these entropy measures under auditory object-specific attention of Rest, AOA1 and AOA2. For example, the mean values of ApEn, SampEn and CmpMSE under auditory object-specific attention of AOA1 are obviously higher than those under auditory object-specific attention of Rest and AOA2. In the viewpoint of mathematics, Figure 1 has a certain intrinsic correlation with Table 2. For instance, for ApEn of P4 channel in Table 2, the *p* values of between Rest and AOA1 and between AOA1 and AOA2 are less than 0.015, which indicate that the differences of the values of ApEn between Rest and AOA1 and between AOA1 and AOA2 are significant. At the same time, in Figure 1 the significant differences of the mean values of ApEn of P4 channel between Rest and AOA1 and between AOA1 and AOA2 are observed. Therefore, the size of the *p* values as shown in Table 2, to a certain extent, can indicate the discriminating power of the entropy measures. The smaller the *p* values, the stronger the discriminating power of the entropy measures may be. As shown in Table 2, the *p* values of CmpMSE, on the whole, are less than those of ApEn and SampEn.

### 3.2. Individual-Level Analysis of Entropy Measures in EEG Signals

**Figure 2:**
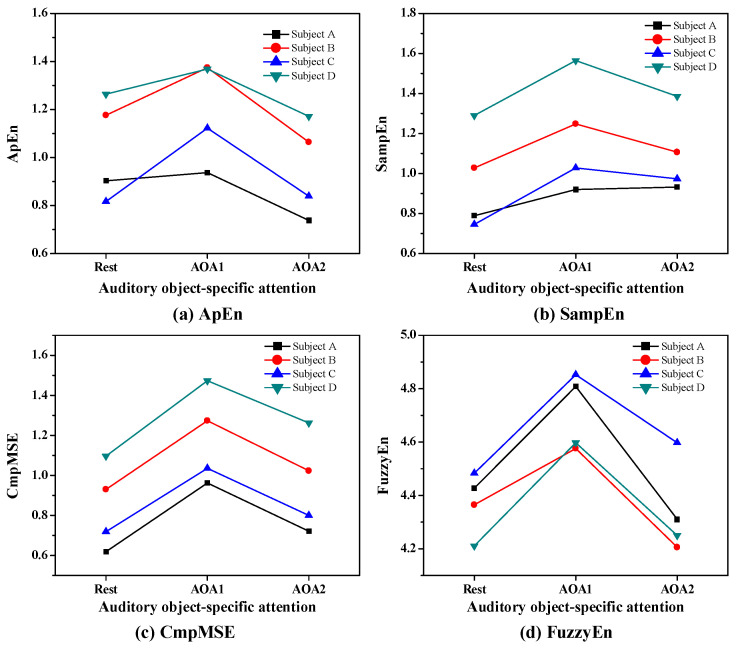
Individual-level analysis of (**a**) ApEn, (**b**) SampEn, (**c**) CmpMSE and (**d**) FuzzyEn in EEG signals of P3 channel under auditory object-specific attention of Rest, AOA1 and AOA2.

To demonstrate the individual-level identification of Rest, AOA1 and AOA2, we first carried out the individual-level analysis of ApEn, SampEn, CmpMSE and FuzzyEn in EEG signals under auditory object-specific attention of Rest, AOA1 and AOA2. Figure 2 presents the values of four subjects’ ApEn, SampEn, CmpMSE and FuzzyEn in EEG signals of P3 channel under auditory object-specific attention of Rest, AOA1 and AOA2 and the EEG signals were selected from four representative subjects.

As shown in Figure 2, it is clearly observed that the values of ApEn, SampEn, CmpMSE and FuzzyEn show obvious differences with respect to Rest, AOA1 and AOA2 of auditory object-specific attention. For the subjects A, B, C and D, the values of ApEn, SampEn, CmpMSE and FuzzyEn on auditory object-specific attention of AOA1 are greater than those of the entropy measures on auditory object-specific attention of Rest and AOA2.

The values of SampEn and CmpMSE on auditory object-specific attention of AOA2 are greater than those of the entropy measures on auditory object-specific attention of Rest, and yet the values of ApEn on auditory object-specific attention of AOA2 are lower than that of ApEn on auditory object-specific attention of Rest.

Through this individual-level analysis of entropy measures on auditory object-specific attention of Rest, AOA1 and AOA2, it was clear that ApEn, SampEn, CmpMSE and FuzzyEn in EEG signals could be used as informative indicators to determine the auditory object-specific attention.

### 3.3. Identification of Auditory Object-Specific Attention by Auditory Attention Classifier

In order to demonstrate the discriminating power with respect to Rest, AOA1 and AOA2 of auditory object-specific attention, the identification of auditory object-specific attention was investigated by two auditory attention classifiers, one used LDA to construct the auditory attention classifier and the other used SVM to construct the auditory attention classifier. The LDA-based auditory attention classifier is designed using a multiclass classification method with open source code [36]. The SVM-based auditory attention classifier is designed using the LIBSVM toolbox [37]. To statistically evaluate whether the identification rates were significantly different from the chance level (33.3%), the chi-squared test was used, with the null hypothesis that the identification rates was dependent of the chance level.

Table 3 shows the identification rates of Rest, AOA1 and AOA2 of auditory object-specific attention by the LDA-based and SVM-based auditory attention classifiers using ApEn, SampEn, CmpMSE and FuzzyEn in EEG signals of eight channels as features. For the LDA-based auditory attention classifier, the average identification rates of the auditory object-specific attention are 48.7%, 46.2%, 43.6% and 46.2%, corresponding to ApEn, SampEn, CmpMSE and FuzzyEn, respectively. For ApEn, SampEn, CmpMSE and FuzzyEn the *p* values of chi-squared test are 0.146, 0.076, 0.079 and 0.076, respectively. For the SVM-based auditory attention classifier, the average identification rates of the auditory object-specific attention are 56.4%, 56.4%, 53.8% and 58.9%, corresponding to ApEn, SampEn, CmpMSE and FuzzyEn, respectively. The corresponding *p* values of chi-squared test are 0.026, 0.026, 0.017 and 0.013, respectively. For the SVM-based auditory attention classifier using ApEn, SampEn, CmpMSE and FuzzyEn as features, the identification rates are significantly different (*p* < 0.05) from the chance level.

As shown in Table 3, on the whole, the identification rates of Rest, AOA1 and AOA2 when using the SVM-based auditory attention classifier are higher than those when using the LDA-based auditory attention classifier. Thus, on the basis of the above results, it is clear that for the identification of auditory object-specific attention the SVM-based auditory attention classifier is more effective than the LDA-based auditory attention classifier.

**Figure 3:**
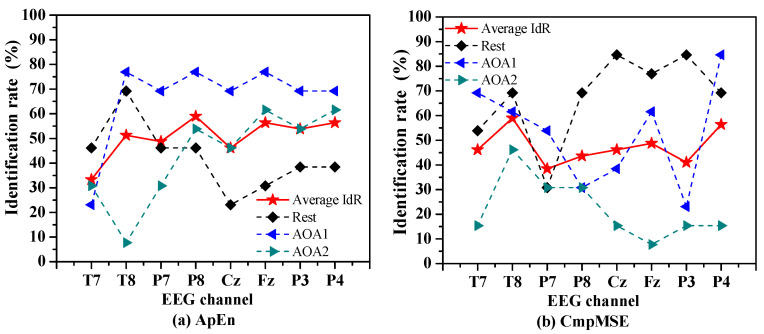
The identification rates of Rest, AOA1 and AOA2 of auditory object-specific attention by the SVM-based auditory attention classifier using (**a**) ApEn and (**b**) CmpMSE in EEG signals per channel as features. The average identification rate (IdR) is calculated by averaging the identification rates of Rest, AOA1 and AOA2. For ApEn the *p* values of chi-squared test are 0.584, 0.001, 0.058, 0.008, 0.048, 0.005, 0.034 and 0.014, which are corresponding to T7, T8, P7, P8, Cz, Fz, P3 and P4, respectively. For CmpMSE the *p* values of chi-squared test are 0.019, 0.012, 0.429, 0.079, 0.003, 0.001, 0.003 and <0.001, which are corresponding to T7, T8, P7, P8, Cz, Fz, P3 and P4, respectively.

To investigate which channel of EEG signals was the most sensitive to the identification of Rest, AOA1 and AOA2 of auditory object-specific attention, we exploited the SVM-based auditory attention classifier to identify the auditory object-specific attention using ApEn and CmpMSE in EEG signals per channel as features. Figure 3 shows the identification rates of Rest, AOA1 and AOA2 by the SVM-based auditory attention classifier using ApEn and CmpMSE in EEG signals per channel as features.

The average identification rate is calculated by averaging the identification rates of Rest, AOA1 and AOA2. For ApEn, the P8, P4 and Fz channels are corresponding to the top three of the average identification rates of auditory object-specific attention and the corresponding identification rates are 59.0% (*p* = 0.008), 56.4% (*p* = 0.014) and 56.4% (*p* = 0.005), respectively. For CmpMSE, the T8, P4 and Fz channels are corresponding to the top three of the average identification rates of auditory object-specific attention and the corresponding identification rates are 59.0% (*p* = 0.012), 56.4% (*p* < 0.001), and 48.7% (*p* = 0.001), respectively. It is clearly observed that for ApEn and CmpMSE the performances of the identification of auditory object-specific attention of Rest, AOA1 and AOA2 vary with the different channels, which may because the different informative features were extracted from different channels by the entropy measures.

**Figure 4:**
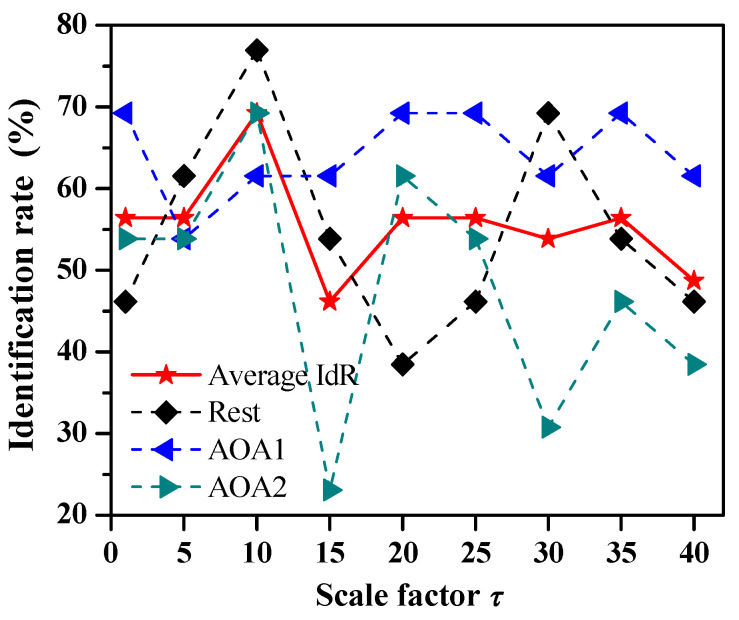
The identification results of auditory object-specific attention by the SVM-based auditory attention classifier using CmpMSE as features. The identification rates of Rest, AOA1 and AOA2 by the adoption of CmpMSE in EEG signals of eight channels with the scale factors *τ* = 1, 5, 10, 15, 20, 25, 30, 35 and 40, and the *p* values of chi-squared test are 0.026, 0.041, <0.001, 0.076, 0.016, 0.026, 0.017, 0.026 and 0.146, respectively.

In order to investigate the influence of the scale factor of entropy measures on the identification rate of auditory object-specific attention, we further studied the identification of auditory object-specific attention of Rest, AOA1 and AOA2 by the SVM-based auditory attention classifier using CmpMSE with different scale factors as features. Figure 4 shows the identification rates of Rest, AOA1 and AOA2 by the adoption of CmpMSE in EEG signals of eight channels with the scale factors *τ* = 1, 5, 10, 15, 20, 25, 30, 35 and 40, respectively. The average identification rate is calculated by averaging the identification rates of Rest, AOA1 and AOA2. The average identification rates are 56.4% (*p* = 0.026), 56.4% (*p* = 0.041), 69.2% (*p* < 0.001), 46.2% (*p* = 0.076), 56.4% (*p* = 0.016), 56.4% (*p* = 0.026), 53.8% (*p* = 0.017), 56.4% (*p* = 0.026) and 48.7% (*p* = 0.146), respectively. Therefore, for CmpMSE the optimal identification of Rest, AOA1 and AOA2 of auditory object-specific attention is achieved with the scale factor *τ* = 10, and the corresponding identification rates of Rest, AOA1 and AOA2 are 69.2%, 76.9% and 61.5%, respectively.

In order to investigate the influence of the choice of parameters for the entropy measures on the identification rate of auditory object-specific attention, we carried out a qualitative comparison of the identification results of auditory object-specific attention by the SVM-based auditory attention classifier using CmpMSE with different parameter values of tolerance. For the calculation of CmpMSE the tolerance was selected to *r* = 0.10, 0.15, 0.20 and 0.25, respectively, and the other parameters were fixed. The qualitative comparison of the identification results of auditory object-specific attention are shown in Table 4. The average identification rates are 59.0% (*p* = 0.020), 69.2% (*p* < 0.001), 71.8% (*p* < 0.001) and 53.8% (*p* = 0.068), corresponding to the tolerance *r* = 0.10, 0.15, 0.20 and 0.25, respectively.

## 4. Discussion

In this study, we explored the entropy measures in EEG signals to extract the informative features relating to auditory object-specific attention and then exploited LDA and SVM to construct the auditory attention classifiers. Our proposed method to the identification of auditory object-specific attention is an innovative attempt. Even though the optimal identification rates of Rest, AOA1 and AOA2 of auditory object-specific attention are only 69.2%, 76.9% and 61.5% respectively by the SVM-based auditory attention classifier using CmpMSE in EEG signals of eight channels with the scale factors *τ* = 10 as features, the identification accuracy is at the same level with the existing studies, for instance, the experimental results reported by Bleichner et al. [38]. We have compared our identification results with the available studies and the comparison results are presented in Table 5.

EEG signals are a kind of non-stationary, non-linear and often multicomponential dynamic signal and it is challenging to accurately extract the informative features of EEG signals. In this study, based on four well-established entropy measures, i.e., ApEn, SampEn, CmpMSE and FuzzyEn, we demonstrate the use of entropy measures in EEG signals as informative features to reveal the auditory attention states. It is clearly shown that the SVM-based auditory attention classifier using ApEn, SampEn, CmpMSE as features are capable of indicating significant differences in informative features of EEG signals under different auditory object-specific attention. This experiment findings are also in line with existing research findings. Many studies also reported that the physiological and cognitive states of human brain could be determined by the use of entropy measures in EEG signals. For example, discrete wavelet transform and entropy measures were used to identify the focal EEG signals [39]; ApEn, SampEn and multiscale entropy were used to assess the different visual attention levels [40]; ApEn was used to evaluate the wakefulness state [25]. These available studies had suggested that the entropy measures of EEG signals, as a complexity parameters of physiological time-series, could be as an useful indicator to reveal the physiological states of human brain and there was no doubt that the entropy measures of EEG signals had clinical significance. Therefore, the entropy measures in EEG signals could also be as informative features to identify the auditory object-specific attention.

When compared with ApEn, SampEn and FuzzyEn, CmpMSE maybe was regarded as the most informative feature of EEG signals to identify the auditory object-specific attention and the optimal identification was achieved by the SVM-based auditory attention classifier using CmpMSE in EEG signals with the scale factor *τ* = 10 and the tolerance *r* = 0.15 and 0.20. This might be because the different entropy measures in EEG signals were able to extract the different informative features of EEG signals. As is known to all, ApEn and SampEn can quantify the temporal structure and complexity of time series strictly at a time scale, usually selected to be 1. But CmpMSE can quantify the long-term structures in EEG signals at multiple time scales, which can extract the long-rang informative feature of EEG signals. The main advantages of our proposed method was that the identification of auditory object-specific attention from single-trial EEG signals was achieved without the need to access to the auditory stimulus, when compared with most available studies. For the existing researches, the auditory attention identification from EEG signals usually required the acoustic features of auditory stimuli be known in advance. The main disadvantages of our proposed method was that the entropy measures were usually computationally intensive and it was necessary to perform comprehensive statistical analyses of the optimal parameters of the computation of the entropy measures and the optimization of machine learning algorithms. However, the algorithm complexity and computing time of CmpMSE are lower than those of ApEn, SampEn and FuzzyEn. Therefore, CmpMSE in EEG signals maybe was the most useful indicator to identify the auditory object-specific attention of Rest, AOA1 and AOA2.

There are some limitations in the current study. Firstly, the 60-s duration of EEG signals used to calculate the entropy measures, corresponding to the data length of *N* = 30,000, is a slightly long time. It is hard to ensure that the EEG signal is stationary or even weakly stationary, which is especially required for permutation entropy analysis [41,42,43]. In fact, we had also evaluated the identification results of auditory object-specific attention by the LDA-based and SVM-based auditory attention classifier using permutation entropy in EEG signals of eight channels as features, and the average identification rates are 23.1% (*p* = 0.429) and 30.8% (*p* = 0.764), respectively. if the EEG signals were split into relatively short epochs and the entropy measures in EEG signals which could be deemed stationary were computed for each of the epochs, and then the distributions of values for each EEG signals were obtained [44,45,46,47], the experimental data would be better to demonstrate the identification results of auditory object-specific attention via entropy measures and machine learning.

Secondly, only 13 subjects participated in the study, and the number of sample data are not enough, which might lead to the statistical results of the entropy measures not showing significance. In fact, this experiment was not very good to perform. In order to ensure the experimental effects for each subject, EEG signals should be recorded successfully in the first round to avoid the second round of experiments because the subject listening to the same auditory stimulus at the second time might cause adverse effects on the auditory attention. Therefore, in the available studies, the number of subjects who participated in the experiment were usually not many. For example, in the studies of Haghighi [13] and Choi [14], ten subjects’ EEG signals were used in the investigations.

Thirdly, for the calculation of entropy measures, it is well-known that some parameters, such as the embedding dimension *m* and tolerance *r*, need to be fixed in advance. We did not assess our experimental performances with the different combinations of the parameters. For one thing, if we such did, it would cause the analysis of experiment results to be rather complex. For another, there were no solid methods to obtain the optimal parameters for the entropy measures. Maybe because of these, the identification rates of Rest, AOA1 and AOA2 of auditory object-specific attention were not very high in this study.

Fourthly, in this study the EEG signals were recorded as 8-channel signals and then the 8-dimensional entropy measures (ApEn, SampEn, CmpMSE and FuzzyEn) could be used to identify the auditory object-specific attention. In Table 3 the number of features of ApEn, SampEn, CmpMSE and FuzzyEn were 8. In Figure 4 and Table 4 the number of features of CmpMSE were 8. We believed that the more EEG signal channels used and the more the dimensionality of entropy measures, the more reliable the experimental results may be, because for the entropy measures the more EEG signal channels were used, more informative features of auditory object-specific attention could be extracted. It also should be noted that, however, the identification rate maybe was not always better with the more EEG signal channels. What’s more, the current research trend was to identify the auditory attention with less EEG signal channels. For example, O’Sullivan et al. used 128-channel EEG signals [7]; Mirkovic et al. used 25-channel EEG signals [48]; Haghighi et al. used 16-channel EEG signals [13]. This is mainly because using less EEG signal channels to identify auditory attention is more worthy research for practical application.

For future work to improve the study presented here, the identification accuracy of auditory object-specific attention maybe has a great potential for improvement. Firstly, we can adopt other advanced machine learning techniques, such as deep learning [49,50,51]. In recent years, deep learning has been widely used in physiological signal application analysis (especially EEG signals), such as seizure prediction [51]. Secondly, we can explore the potential of other non-linear feature analysis methods of EEG signals for the identification of auditory object-specific attention, such as higher order spectra [52], phase entropy [53], wavelet transform in conjunction with entropy [39], empirical mode decomposition in conjunction with entropy [54] and so on. In addition, we can adopt several different types of features of EEG signals in conjunction with feature ranking approach [53,55] to further investigate the identification of auditory object-specific attention.

The identification of auditory object-specific attention would undoubtedly have great research value and application potential for the optimization of hearing aids and enhanced listening techniques, which are our main clinical application. For example, the algorithm of identification of auditory object-specific attention would work hand in hand with the algorithms of acoustic scene analysis in hearing aids to form neuro-steered hearing prostheses. With the help of the identification of peoples’ attention to a specific auditory object from EEG signals, we can use EEG signals to guide the algorithms of acoustic scene analysis, in effect extending the efferent neural pathways which simulates the top-down cognitive control of auditory attention.

## 5. Conclusions

In this paper, ApEn, SampEn, CmpMSE and FuzzyEn were used to extract the informative features of EEG signals under three kinds of auditory object-specific attention (Rest, AOA1 and AOA2). The results of statistical analysis of entropy measures indicated that there were significant differences (*p* < 0.05) in the values of ApEn, SampEn, CmpMSE and FuzzyEn in EEG signals under auditory object-specific attention of Rest, AOA1 and AOA2. LDA and SVM were used to construct the auditory attention classifiers respectively and LOOCV was used to evaluate the identification rates of Rest, AOA1 and AOA2 of auditory object-specific attention. Compared with the LDA-based auditory attention classifier, the SVM-based auditory attention classifier was capable of achieving better auditory object-specific attention identification accuracy for Rest, AOA1 and AOA2. According to the identification results, for Rest, AOA1 and AOA2 of auditory object-specific attention, the optimal identification was achieved by the SVM-based auditory attention classifier using CmpMSE with the scale factor *τ* = 10 and the corresponding identification rates were 69.2%, 76.9% and 61.5%, respectively. All results suggest that using the entropy measures in EEG signals as informative features in conjunction with machine learning techniques can provide a novel solution to the identification of auditory object-specific attention from single-trial EEG signals without the need to access to the auditory stimulus.

## Figures and Tables

**Table 1:** The assignment of the parameters for the calculation of approximate entropy (ApEn), sample entropy (SampEn), composite multiscale entropy (CmpMSE) and fuzzy entropy (FuzzyEn). STD is denoted as the standard deviation of electroencephalography (EEG) signals.

Entropy Measures	Data Length	Time Delay	Embedding Dimension	Tolerance	Scale Factor	Reference
	*N*	*t*	*m*	*r*	*τ*	
ApEn	30,000	1	2	0.15 × STD	−	[27]
SampEn	30,000	1	2	0.15 × STD	−	[27]
CmpMSE	30,000	1	2	0.15 × STD	30	[28]
FuzzyEn	30,000	1	2	0.15 × STD	−	[30]

**Table 2:** The *p* values of multiple comparisons between the auditory object-specific attention of Rest, AOA1 and AOA2 for ApEn, SampEn and CmpMSE.

		T7	T8	P7	P8	Cz	Fz	P3	P4
ApEn	Rest vs. AOA1	0.382	0.015	0.078	0.041	0.035	0.024	0.011	0.014
	Rest vs. AOA2	0.260	0.793	0.936	0.430	0.296	0.109	0.650	0.999
	AOA1 vs. AOA2	0.015	0.003	0.036	0.001	0.001	0.001	0.001	0.013
SampEn	Rest vs. AOA1	0.046	0.001	0.001	0.002	−	0.013	−	−
	Rest vs. AOA2	0.777	0.256	0.021	0.660	−	0.430	−	−
	AOA1 vs. AOA2	0.183	0.042	0.027	0.023	−	0.246	−	−
CmpMSE	Rest vs. AOA1	0.034	0.002	0.005	0.002	0.002	0.010	0.001	0.001
	Rest vs. AOA2	0.413	0.216	0.182	0.318	0.296	0.800	0.107	0.191
	AOA1 vs. AOA2	0.393	0.122	0.268	0.071	0.093	0.058	0.204	0.025
FuzzyEn	Rest vs. AOA1	0.001	0.001	−	0.001	0.001	0.001	0.001	0.001
	Rest vs. AOA2	0.051	0.050	−	0.047	0.038	0.045	0.021	0.016
	AOA1 vs. AOA2	0.249	0.138	−	0.138	0.001	0.004	0.002	0.008

**Table 3:** The identification rates of Rest, AOA1 and AOA2 of auditory object-specific attention by the linear discriminant analysis-based (LDAb) and support vector machine-based (SVMb) auditory attention classifiers (AAC) using ApEn, SampEn, CmpMSE and FuzzyEn in EEG signals as features. The *p* values indicate the level of significance between the identification rates and chance level (33.3%). LDAb AAC, LDA-based auditory attention classifier; SVMb AAC, SVM-based auditory attention classifier; Average IdR, average identification rate.

Identification Rate		ApEn	SampEn	CmpMSE	FuzzyEn
LDAb AAC	Rest (%)	38.5	38.5	30.8	53.9
	AOA1 (%)	61.5	69.2	69.2	61.5
	AOA2 (%)	46.2	30.8	30.8	23.1
	Average IdR (%)	48.7	46.2	43.6	46.2
	*p* value	0.146	0.076	0.079	0.076
SVMb AAC	Rest (%)	53.8	46.2	69.2	69.2
	AOA1 (%)	69.2	69.2	61.5	61.5
	AOA2 (%)	46.2	53.8	30.8	46.2
	Average IdR (%)	56.4	56.4	53.8	58.9
	*p* value	0.026	0.026	0.017	0.013

**Table 4:** The qualitative comparison of the identification results of auditory object-specific attention by the SVM-based auditory attention classifier using CmpMSE with different parameter values of tolerance. For the calculation of CmpMSE the tolerance was selected to *r* = 0.10, 0.15, 0.20 and 0.25, respectively and the other parameters were fixed with the data length *N* = 30,000, the embedding dimension *m* = 2 and the scale factor *τ =* 10.

Tolerance *r*	Identification Rate (%)			Average IdR (%)
	Rest	AOA1	AOA2	
0.10	61.5	61.5	53.8	59.0
0.15	69.2	76.9	61.5	69.2
0.20	69.2	76.9	69.2	71.8
0.25	53.8	61.5	46.2	53.8

**Table 5:** The comparison of our identification results with the existing studies for the identification of auditory attention based on EEG signals.

References	Auditory Task	Number of Channels	Identification Approaches	Identification Results
[38]	The direction of attention (front, left and right)	84	Leave-one-out cross validation template matching approach	70%
[14]	Attending left and attending right	32	ERP templates; Leave-one-out cross-subject validation	71.2%
Our work	Auditory object-specific attention (Rest, AOA1 and AOA2)	8	Composite multiscale entropy; Leave-one-out cross validation	69.2%, 76.9% and 61.5%
